# Evaluation of Concurrent Chemoradiotherapy for Survival Outcomes in Patients With Synchronous Oligometastatic Esophageal Squamous Cell Carcinoma

**DOI:** 10.1001/jamanetworkopen.2022.44619

**Published:** 2022-12-01

**Authors:** Zhenguo Shi, Xiaojuan Zhu, Changli Ruan, Gang Wei, Jiaojiao Li, Hu Qiu, Lijuan Gao, Gaoke Cai, Yutian Zhangcai, Bin Li, Jing Wang, Yi Gong, Jiamei Chen, Wensi Zhao, Yong Wu, Shaobo Ke, Yongshun Chen

**Affiliations:** 1 Department of Clinical Oncology, Renmin Hospital of Wuhan University, Wuhan, Hubei Province, People's Republic of China; 2 Department of Oncology, The First Affiliated Hospital of Henan University of Science and Technology, Luoyang, Henan Province, People's Republic of China

## Abstract

**Importance:**

The optimal treatment for and potential benefit populations of synchronous oligometastatic esophageal squamous cell carcinoma (SOESCC) remain unclear.

**Objectives:**

To evaluate outcomes of concurrent chemoradiotherapy (CCRT) and to construct decision tree models for predicting the risk of progression and mortality in patients with SOESCC.

**Design, Setting, and Participants:**

This prognostic study included 532 patients with SOESCC who were treated at 2 cancer centers in China from January 2012 to December 2018 and consisted of a development cohort (n = 381) and a validation cohort (n = 151). Data were analyzed from March 2019 to December 2021.

**Exposures:**

All patients received chemotherapy alone or CCRT.

**Main Outcomes and Measures:**

The primary end points of the study were progression-free survival (PFS) and overall survival (OS), and the secondary end points were locoregional control and treatment-related toxic effects. Propensity score matching was performed to control potential confounding factors. Cox regression was used to screen important explanatory variables. Decision trees for optimally partitioning patients were established using recursive partitioning analysis and were then subjected to internal and independent external validation.

**Results:**

Among the 532 patients (median [range] age, 63 [32-82] years; 367 men [69.0%]), 292 patients received chemotherapy alone and 240 patients underwent CCRT. With a median (IQR) follow-up time of 37.0 (21.6-55.8) months, CCRT was associated with improved objective response rate (139 of 240 [57.9%] vs 123 of 292 [42.1%]; *P* < .001), median (IQR) PFS (9.7 [8.5-10.9] months vs 7.6 [6.6-8.6] months; *P* < .001), and median (IQR) OS (18.5 [16.1-20.9] months vs 15.2 [13.6-16.8] months; *P* < .001) compared with chemotherapy alone. Propensity score matching analysis verified the results. Cox multivariate analysis indicated that treatment modality (CCRT vs chemotherapy alone) was an independent prognostic factor related to PFS (hazard ratio, 0.69; 95% CI, 0.57-0.83; *P* < .001) and OS (hazard ratio, 0.75; 95% CI, 0.61-0.93; *P* = .008). The final decision trees divided patients with SOESCC into low-, intermediate-, and high-risk groups in both the internal and external validations, and the corresponding cumulative risk function curves had significant differences (all *P* < .001). Time-dependent maximum areas under receiver operating curves of decision trees for progression risk at 3 years and mortality risk at 5 years were 0.820 (95% CI, 0.693-0.948) and 0.894 (95% CI, 0.822-0.966), respectively. Calibration curves also demonstrated that the decision trees had favorable performance of risk stratification.

**Conclusions and Relevance:**

In this study, CCRT vs chemotherapy alone as a first-line treatment for patients with SOESCC had superior survival. Patients with low risk had promising long-term survival based on the current treatment modality. The predictive information of the decision tree could provide accurate decision-making for the management of patients with SOESCC.

## Key Points

**Question:**

Should concurrent chemoradiotherapy be considered for patients with synchronous oligometastatic esophageal squamous cell carcinoma (SOESCC), and which patients are suitable for early intensive treatment?

**Findings:**

In this prognostic study of 532 patients with SOESCC, concurrent chemoradiotherapy was associated with significant improvements in survival outcomes compared with chemotherapy alone. Decision trees that optimally partitioned patients were developed and validated using recursive partitioning analysis based on the clinical parameters affecting survival.

**Meaning:**

The decision trees created in this study could be used to identify patients with SOESCC who would benefit most from early individualized treatment.

## Teaser

This prognostic study evaluates the outcomes of concurrent chemoradiotherapy and constructs decision tree models for predicting the risk of progression and mortality in patients with synchronous oligometastatic esophageal squamous cell carcinoma (ESCC).

## Introduction

Esophageal cancer (EC) is the seventh most common malignant tumor, and overall mortality ranks sixth worldwide.^1^ The geographical difference in incidence rate and prognosis of EC is significantly different between the 2 most common histological subtypes: squamous cell carcinoma and adenocarcinoma.^2^ Esophageal squamous cell carcinoma (ESCC) accounts for 70% of global EC cases and is the main EC subtype in the Asian population.^3,4^ Despite multidisciplinary treatment, the overall 5-year survival rate of patients with EC ranges from 15% to 25%.^5^ Preoperative concurrent chemoradiotherapy (CCRT) or definitive CCRT should be offered to patients with locally advanced ESCC, but definitive CCRT is an option for patients who cannot tolerate or choose not to undergo surgery.^6^

According to the European Society for Radiotherapy and Oncology (ESTRO) and American Society for Radiation Oncology (ASTRO) consensus document, oligometastatic disease (OMD) has been defined as 1 to 5 metastatic lesions that are safely treatable, with or without a controlled primary tumor.^7^ Furthermore, the consensus of ESTRO and the European Organization for Research and Treatment of Cancer (EORTC) suggests that patients with OMD can be divided into synchronous OMD (maximum of 6-month interval between diagnosis of OMD and primary cancer diagnosis) and metachronous OMD (more than 6-month interval between diagnosis of OMD and primary cancer diagnosis).^8^

Oligometastatic EC, the intermediate phase before extensive metastasis, does not quickly progress to a widespread distribution of cancer and can benefit from the multidisciplinary team approach.^9^ At present, there is no specific set of uniform standards for CCRT in patients with synchronous oligometastatic ESCC (SOESCC). Our previous study^10^ suggested that CCRT can provide preferable progression-free survival (PFS) and overall survival (OS) in older patients with SOESCC compared with chemotherapy alone.^10^ However, given the rapid progression in a certain proportion of patients after initial treatment, reasonable stratification can improve the recognition of the biological behavior of oligometastatic EC, and screen out the subgroups suitable for early intensive treatment. Therefore, this study was designed to evaluate the outcomes associated with CCRT for patients with SOESCC and to construct decision tree models predicting the risk of progression and mortality with recursive partitioning analysis.

## Methods

### Patients

This prognostic study included the database of patients who were treated at Renmin Hospital of Wuhan University and The First Affiliated Hospital of Henan University of Science and Technology from January 2012 to December 2018. The institutional review board of Renmin Hospital of Wuhan University approved this study. Informed consent was exempt given the retrospective nature of the study. This study followed the Transparent Reporting of a Multivariable Prediction Model for Individual Prognosis or Diagnosis (TRIPOD) reporting guideline^11^ and adhered to the Declaration of Helsinki.^12^

We adopt the definition of synchronous OMD according to the consensus reached by ESTRO-EORTC in 2020.^8^ The inclusion criteria were as follows: (1) age 15 to 85 years; (2) Eastern Cooperative Oncology Group (ECOG) performance status (PS) score 0 to 2; (3) histology and pathology confirmed ESCC; (4) tumor-node-metastasis stage according to American Joint Committee on Cancer seventh edition (2009); (5) 5 or fewer measurable metastatic lesions; (6) synchronous oligometastasis present (ie, interval time from primary cancer diagnosis to OMD of ≤6 months); (7) chemotherapy regimen consisted of cisplatin plus 5-fluorouracil or docetaxel; and (8) no previous radiotherapy, chemotherapy, and other tumor-related treatments. The exclusion criteria were as follows: (1) esophageal adenocarcinoma; (2) incomplete CCRT; (3) incomplete follow-up data; or (4) combined perforation, hemorrhage, or infectious diseases before treatment.

Baseline characteristics were assessed for all patients, including endoscopy, hematology test, enhanced computed tomography, whole-body bone scan, and positron emission tomography–computed tomography (if necessary). The patients were then divided into the chemotherapy-alone group and the CCRT group.

### Chemotherapy Alone

For the cisplatin/5-fluorouracil regimen, cisplatin (25 mg/m^2^) was infused intravenously from day 1 to day 3 and fluorouracil (500 mg/m^2^) from day 1 to day 5. For cisplatin/docetaxel regimen, an intravenous infusion of cisplatin (25 mg/m^2^) from day 1 to day 3 and docetaxel (75 mg/m^2^) on day 1 was applied. The chemotherapy regimen was performed and repeated every 4 weeks until disease progression, intolerable toxic effects, or patient’s refusal to continue.

### CCRT

External irradiation was administered with a high-energy (≥6 megavolt) linear accelerator, and all patients received intensity-modulated radiation therapy, with 5 fractions per week. Clinical target volume (CTV) was defined as the primary tumor plus 2 cm superiorly and inferiorly along the length of the esophagus and a 1-cm radial expansion, and the total radiation dose was 50Gy/25 fractions. The CTV of metastatic lesion was established by a 1-cm expansion around the metastatic tumor, and a dose of 45 Gy/18 fractions was delivered. All planning target volume was defined on intensity-modulated radiation therapy by adding a 0.5-cm margin to the CTV. The radiation dose reduction strategies were recommended for metastatic lesions at special sites, such as adjacent to intestine or spinal cord, and the irradiation of metastatic lesions was concurrent with primary tumor. The same chemotherapy regimen was performed and repeated every 4 weeks throughout the radiotherapy period. The regimen was then continued until disease progression, intolerable toxic effects, or patient’s refusal to continue.

### Outcomes

The primary end points of the present study were PFS and overall survival OS, and the secondary end points were locoregional control and treatment-related toxic effects. PFS was defined as the time from first treatment to disease progression or mortality from any cause. OS was measured from the first day of treatment to death or the last follow-up. The tumor response of the primary tumor and metastatic lesions was evaluated according to the Response Evaluation Criteria in Solid Tumors (RECIST) version 1.1,^13^ as follows: complete response (CR), partial response (PR), stable disease (SD), and progressive disease (PD). The objective response rate (ORR) included CR and PR. The disease control rate (DCR) included CR, PR, and SD. Treatment-associated adverse events were graded by National Cancer Institute’s Common Terminology Criteria for Adverse Events (version 4.0) and the Radiation Therapy Oncology Group (RTOG) criteria.

### Statistical Analysis

We used the χ^2^ test or Fisher exact test to compare categorical variables between the CCRT and chemotherapy-alone groups. For continuous data, the *t* test and Mann-Whitney *U* test were performed, depending on the normality of the data. Median follow-up was determined using the reverse Kaplan-Meier estimator. Rates of PFS and OS were estimated by the Kaplan-Meier method, and the log-rank test was used to compare the differences between groups. The prognostic analysis of PFS and OS adopts univariate and multivariate Cox proportional hazards regression model. All factors associated with PFS and OS were included in multivariate Cox regression analysis to test their association with potential predictors, regardless of the significance level on univariate analysis. Results were reported as hazard ratios (HR) and 95% CI. A 2-tailed *P* < .05 was considered statistically significant.

Propensity score matching (PSM) analysis was performed to further compare the outcomes of CCRT and chemotherapy alone. One-to-one matching without replacement was completed on the logit of the propensity score by using the nearest-neighbor match. Caliper width was 0.05 times the standard deviation of the logit of propensity score, which can eliminate more than 99% of the deviation due to confounding variables.^14^ The standardized absolute mean differences were estimated for all baseline covariates before and after matching to evaluate the imbalance between treatment groups, and a standardized absolute mean difference of less than 0.1 for a given covariate suggested a good performance of the propensity score.^15^

To further account for disease burden, using the decision tree, a recursive partitioning analysis was used to divide the whole patient cohort into subgroups according to the clinical factors associated with PFS and OS screened by univariate Cox regression in the development cohort (from Renmin Hospital of Wuhan University). For a given group of patients (or cluster in recursive partitioning analysis), the algorithm recursively iterates each binary partition of these patients according to the available factors. The binary partition that produced the most statistically significant difference in OS and PFS was used to divide the cluster into 2 subclusters, and the process was repeated. The significance level was adjusted for iterative multiple comparisons. To define the optimal decision tree size, the complexity parameter (CP) was executed as the penalty factor to control the tree size. Within the range of the corresponding standard deviation of the minimum cross-validation error, the minimum CP value was selected to prune the tree, and finally, the terminal node of the decision tree was determined and marked as the corresponding risk level of progression or mortality.^16^

For internal validation, the corresponding cumulative risk function curves were drawn by fitting the survival function of patients with different risk levels, and the log-rank test was used to verify the prognostic value of the decision tree. Meanwhile, the time-dependent receiver operating characteristic (ROC) curves were generated, and the area under the curve (AUC) was performed to assess the discrimination ability of the decision tree.^17^ The decision tree was subjected to 1000 bootstrap resamples for internal cross-validation of the primary development cohort. Calibration was quantified by comparing the actual observed progression or mortality risk with the model-predicted progression or mortality risk, and the results were graphically evaluated as calibration curves. Perfect calibration would be exhibited by a direct alignment between the actual observation and decision tree prediction probability along the 45° diagonal line. The decision tree models were then tested in the independent external validation cohort (from the First Affiliated Hospital of Henan University of Science and Technology) using the same method. SPSS statistical software version 22.0 (IBM Corp) and R version 4.0.3 (R Project for Statistical Computing) were used for statistical analysis. Data were analyzed from March 2019 to December 2021.

## Results

A total of 532 patients with SOESSC were analyzed with a median age of 63 years (range 32-82 years), consisting of 381 patients from the Renmin Hospital of Wuhan University and 151 patients from the First Affiliated Hospital of Henan University of Science and Technology. Of the entire cohort, most patients were male (367 [69.0%]), and primary tumor length was 5 cm or greater (321 [60.3%]). Overall, 289 patients (54.3%) had tumors located in the middle thoracic esophagus. A total of 321 patients (60.3%) had 1 metastatic organ, and 211 (39.7%) had 2 to 3; 308 patients (57.9%) had 1 to 3 metastatic lesions, and 224 (42.1%) had 4 to 5. Overall, 292 patients received chemotherapy alone, and 240 patients underwent CCRT. The baseline clinical characteristics of patients are summarized in the Table.

**Table.** Characteristics of 532 Patients

Characteristic	Patients by treatment modality, No. (%)		*P* value
	Chemotherapy alone (n = 292)	CCRT (n = 240)	
Age, y			
<60	105 (36.0)	71 (29.6)	.12
≥60	187 (64.0)	169 (70.4)	
Median (range)	62.5 (35.0-82.0)	64.0 (32.0-81.0)	.08
Sex			
Male	211 (72.3)	156 (65.0)	.07
Female	81 (27.7)	84 (35.0)	
ECOG PS			
0	25 (8.6)	24 (10.0)	.31
1	221 (75.7)	185 (77.1)	
2	46 (15.8)	31 (12.9)	
Primary tumor length			
<5 cm	126 (43.2)	85 (35.4)	.07
≥5 cm	166 (56.8)	155 (64.6)	
Primary tumor location			
Cervical	11 (3.8)	18 (7.5)	.001
Upper	33 (11.3)	54 (22.5)	
Middle	170 (58.2)	119 (49.6)	
Lower	78 (26.7)	49 (20.4)	
Site of metastasis			
Distant lymph node	243 (83.2)	207 (86.3)	.06
Lung	105 (36.0)	70 (29.2)	
Bone	33 (11.3)	31 (12.9)	
Liver	57 (19.5)	25 (10.4)	
Adrenal gland	1 (0.3)	2 (0.8)	
No. of metastatic organs			
1	164 (56.2)	157 (65.4)	.03
2-3	128 (43.8)	83 (34.6)	
No. of metastatic lesions			
1-3	154 (52.7)	154 (64.2)	.008
4-5	138 (47.3)	86 (35.8)	
Chemotherapy regimen			
DP	146 (50.0)	115 (47.9)	.63
PF	146 (50.0)	125 (52.1)	
Radiation dose, primary tumor/metastasis, Gy			
50/45	0	218 (90.8)	NA
40-46/30-45	0	22 (9.2)	

Abbreviations: CCRT, concurrent chemoradiotherapy; DP, cisplatin plus docetaxel; ECOG, Eastern Cooperative Oncology Group; NA, not applicable; PS, performance status; PF, cisplatin plus 5-fluorouracil.

### Tumor Responses

**Figure 1.**
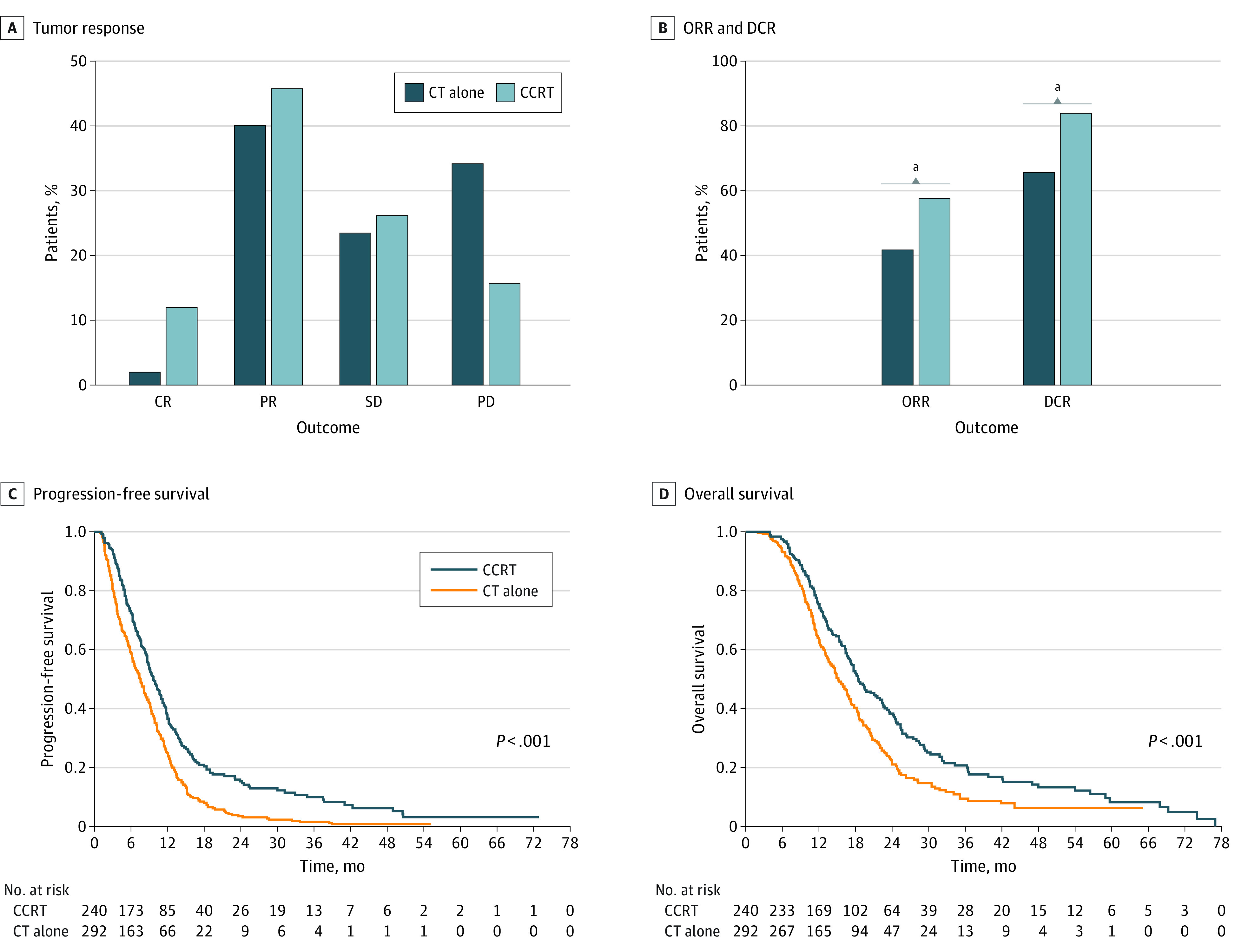
Oncological Outcomes of the Treatment Modality CR indicates complete response; CCRT, concurrent chemoradiotherapy; CT, chemotherapy; DCR, disease control rate; ORR, objective response rate; PD, progressive disease; PR, partial response; SD, stable disease. ^a^*P* < .001

All patients in this cohort were evaluated for tumor responses. In the chemotherapy-alone group, only 6 patients (2.1%) achieved CR, 117 patients (40.1%) had PR, 69 patients (23.6%) exhibited SD, and 100 patients (34.2%) had PD. By comparison, in the CCRT group, 29 patients (12.1%) had CR, 110 patients (45.8%) had PR, 63 patients (26.3%) had SD, and only 38 patients (15.8%) experienced PD (Figure 1A). CCRT was associated with significant improvements in ORR and DCR compared with chemotherapy alone (ORR: 139 of 240 [57.9%] vs 123 of 292 [42.1%]; *P* < .001; DCR: 202 of 240 [84.2%] vs 192 of 292 [65.8%]; *P* < .001) (Figure 1B).

### Survival Outcomes Before PSM

For all patients, the median (IQR) follow-up time was 37.0 (21.6-55.8) months. During the whole study period, 271 patients (92.8%) in the chemotherapy-alone group and 207 (86.2%) in the CCRT group experienced disease progression, and the corresponding median PFS was 7.6 (95% CI, 6.6-8.6) months and 9.7 (95% CI, 8.5-10.9) months, respectively (*P* < .001) (Figure 1C). The median OS was 15.2 (95% CI 13.6-16.8) months for the chemotherapy-alone group and 18.5 (95% CI, 16.1-20.9) months for the CCRT group (*P* < .001) (Figure 1D).

Multivariate Cox regression analysis found that treatment modality (HR, 0.69; 95% CI, 0.57-0.83; *P* < .001), age (HR, 0.72; 95% CI, 0.59-0.87; *P* = .001), and ECOG PS of 1 (HR, 1.85; 95% CI, 1.31-2.60; *P* < .001) or 2 score (HR, 1.78; 95% CI, 1.18-2.68; *P* = .006) were associated with PFS (eFigure 1 in Supplement 1). Meanwhile, treatment modality (HR, 0.75; 95% CI, 0.61-0.93; *P* = .008), age (HR, 0.72; 95% CI, 0.58-0.89; *P* = .003), the number of metastatic lesions (HR, 1.49; 95% CI, 1.02-2.19; *P* = .04), and ECOG PS of 1 (HR, 2.54; 95% CI, 1.69-3.83; *P* < .001) or 2 score (HR, 2.87; 95% CI, 1.78–4.61; *P* < .001) were associated with OS (eFigure 2 in Supplement 1).

### Survival Outcomes After PSM

**Figure 2.**
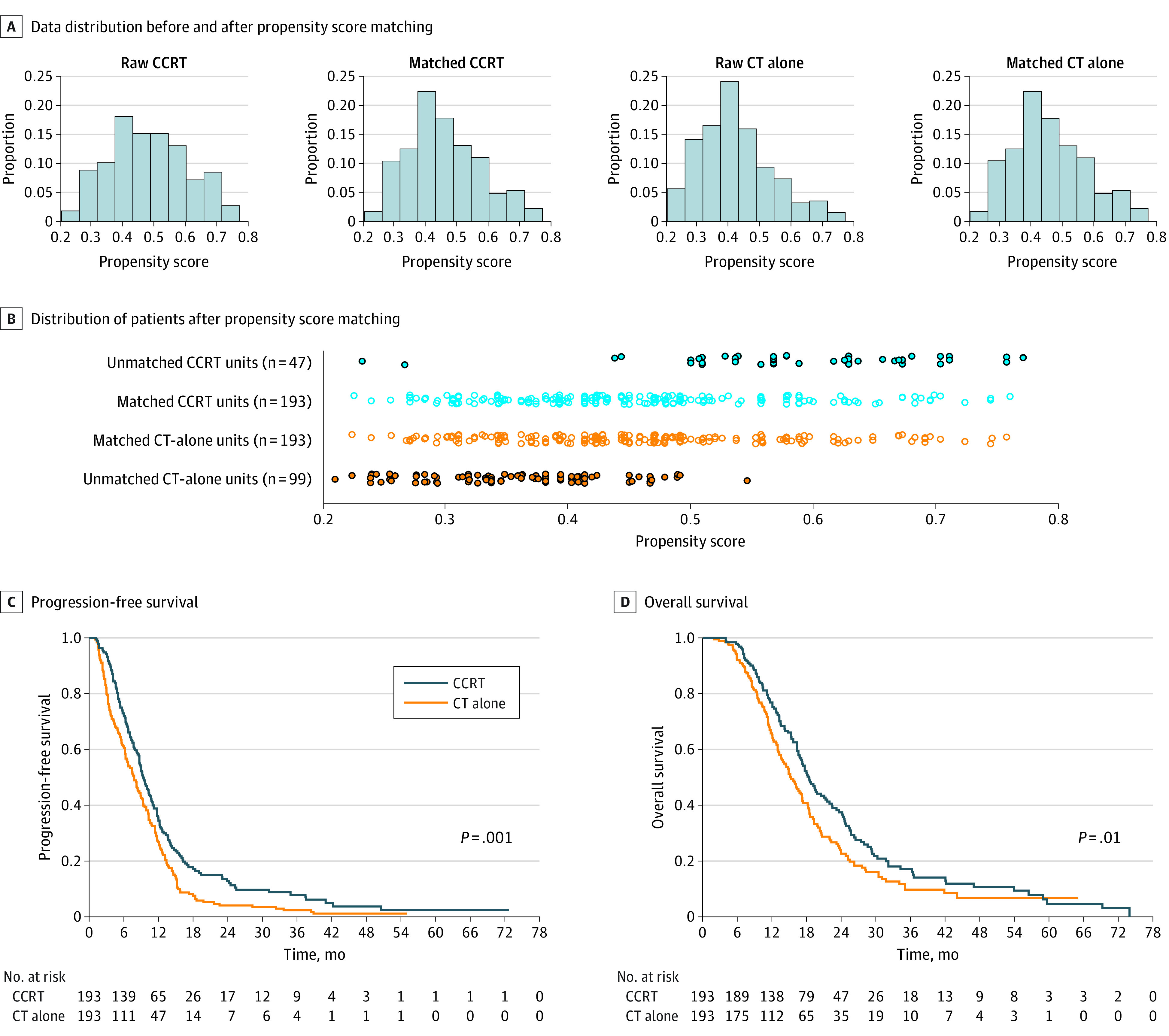
Propensity Score Matching and Survival CCRT indicates concurrent chemoradiotherapy; CT, chemotherapy.

The distribution of patients before and after PSM is shown in Figure 2A; 193 patients with SOESCC who received chemotherapy alone and 193 patients who underwent CCRT were matched (Figure 2B). The baseline characteristics of patients were well balanced in all covariates (eFigure 3 and eTable 1 in Supplement 1). The median PFS was 7.8 (95% CI, 6.4-9.2) months in the chemotherapy-alone group and 9.4 (95% CI, 8.3-10.5) months in the CCRT group (*P* = .001) (Figure 2C). The median OS was 15.3 (95% CI, 13.3-17.3) months for the chemotherapy-alone group and 18.4 (95% CI, 16.7-20.1) months for the CCRT group (*P* = .012) (Figure 2D).

### Decision Trees

The development cohort enrolled 381 eligible patients (eTable 2 in Supplement 1). The clinical factors associated with PFS were age, ECOG PS, tumor location, number of metastatic organs, number of metastatic lesions, treatment modality, and tumor response. Factors found to be significantly associated with OS included age, ECOG PS, number of metastatic organs, number of metastatic lesions, treatment modality, and tumor response (eTable 3 in Supplement 1). These factors were used to generate the best decision tree.

**Figure 3.**
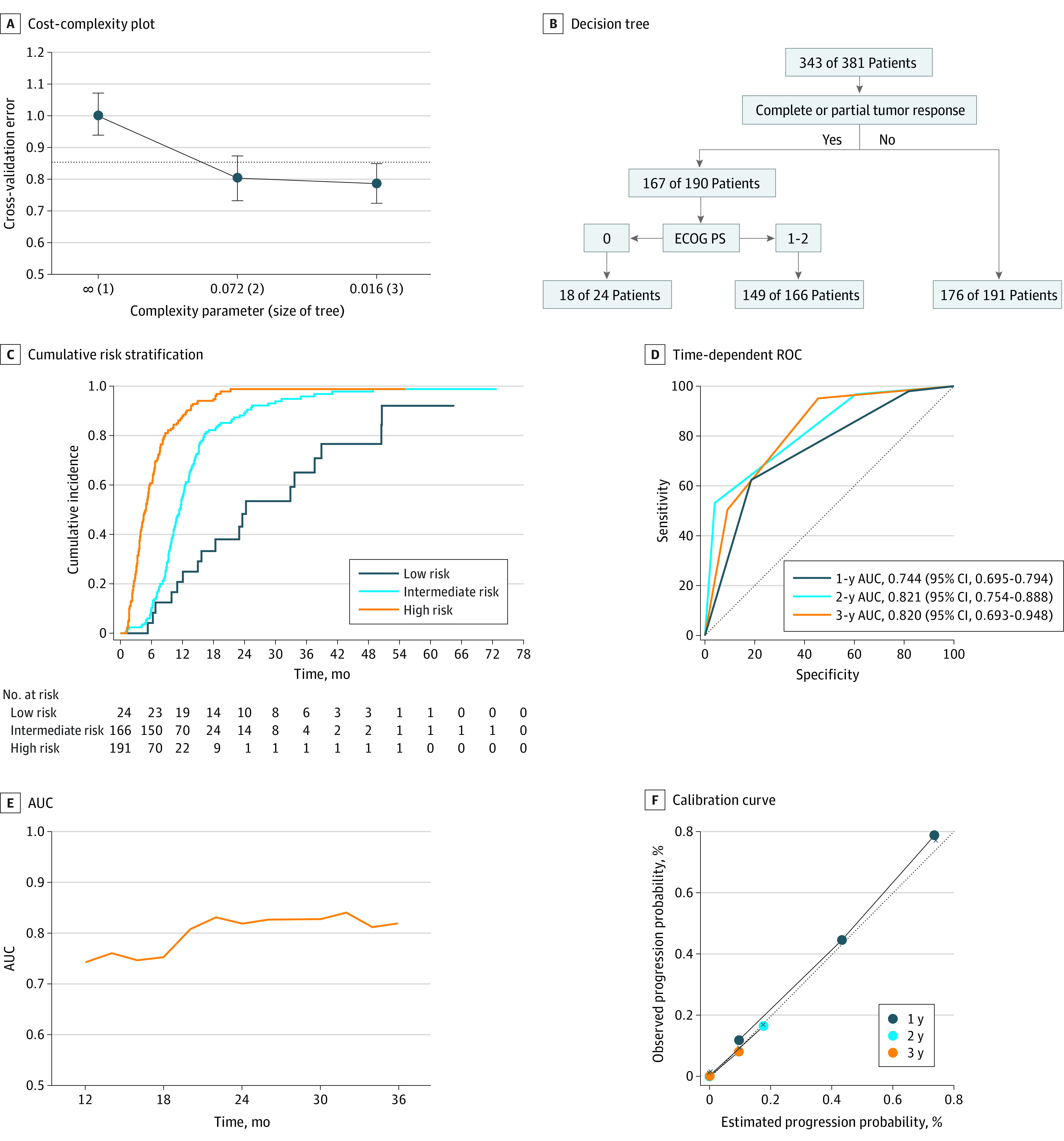
Progression Risk Stratification of Decision Tree and Internal Validation in the Development Cohort AUC indicates area under the curve; ECOG PS, Eastern Cooperative Oncology Group Performance Status; ROC, receiver operating characteristic curves. F, Dotted line indicates perfect calibration.

To build a decision tree model for progression risk, the cross-validation error was used to determine the optimal level of tree complexity. The minimum CP corresponding to a standard deviation range of the minimum cross-validation error was 0.016 (Figure 3A). The final decision tree after pruning divided patients with SOESCC into low-, intermediate-, and high-risk groups (Figure 3B; eTable 4 in Supplement 1), and the corresponding cumulative risk function curves were drawn by fitting the survival function of patients with different risk levels (*P* < .001) (Figure 3C). The time-dependent ROC (1-year AUC, 0.744; 95% CI, 0.695-0.794); 2-year AUC, 0.821; 95% CI, 0.754-0.888; 3-year AUC, 0.820; 95% CI, 0.693-0.948) and AUC for predicting progression risk are shown in Figures 3D and E. The calibration curve of predicted probability and actual observed events approach a 45° diagonal (Figure 3F).

**Figure 4.**
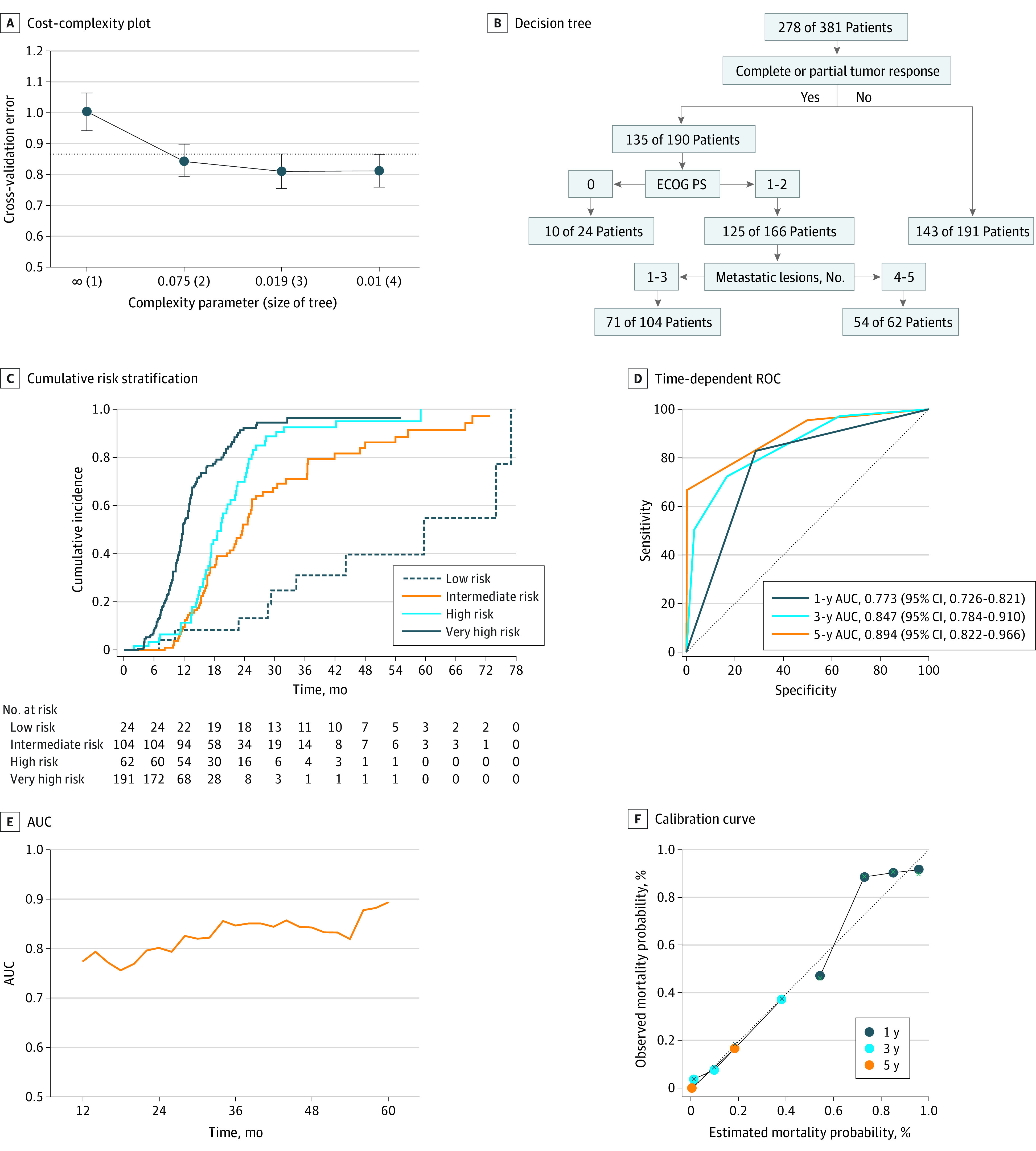
Mortality Risk Stratification of Decision Tree and Internal Validation in the Development Cohort AUC indicates area under the curve; ECOG PS, Eastern Cooperative Oncology Group Performance Status; ROC, receiver operating characteristic curves. F, Dotted line indicates perfect calibration.

The same method was used to develop the final decision tree model for mortality risk. After a minimum CP of 0.01 (Figure 4A), patients were classified into low-, intermediate-, high-, and very high-risk groups according to the final decision tree (Figure 4B; eTable 5 in Supplement 1), and the corresponding cumulative risk function curves were drawn (*P* < .001) (Figure 4C). The time-dependent ROC (1-year AUC, 0.773; 95% CI, 0.726-0.821; 3-year AUC, 0.847; 95% CI, 0.784-0.910; 5-year AUC, 0.894; 95% CI, 0.822-0.966) and AUC of the predicted mortality risk are displayed in Figures 4D and E. The model was well calibrated (Figure 4F).

The independent external validation cohort included 151 eligible patients (eTable 2 in Supplement 1). All patients were well classified into different risk groups of progression (eFigure 4A in Supplement 1) and mortality (eFigure 4B in Supplement 1). The calibration curves demonstrated good performance of the decision trees (eFigure 4C and D in Supplement 1).

### Treatment-Related Toxic Effects

The most frequent treatment-related toxic effects were leukocytopenia, nausea, and vomiting. Rates of grade 3 and 4 leukocytopenia were 16.1% (47 patients) and 6.2% (18 patients), respectively, in the chemotherapy-alone group and 21.3% (51 patients) and 9.6% (23 patients), respectively, in the CCRT group (*P* = .03). Fatigue and alanine aminotransferase level elevation were also commonly observed. Compared with chemotherapy alone, other major grade 3 or greater treatment-related toxic effects included radiation pneumonitis (16 patients [6.7%]) and radiation esophagitis (17 [7.1%]) for the CCRT group. Treatment was well tolerated in both groups, and there were no treatment-related deaths (eTable 6 in Supplement 1).

## Discussion

Initially, we found a significant association of CCRT for primary tumors and metastatic lesions with PFS and OS from the largest cohort of patients with SOESCC of which we are aware. This association was still statistically significant in Cox regression analysis, and PSM analysis reconfirmed the association. These findings suggested that the combination of aggressive chemotherapy and radiotherapy could improve the outcomes in selected patients with SOESCC compared with chemotherapy alone. Importantly, patients were divided into different risk levels based on the decision tree model, which will help to guide future research in the field of SOESCC.

RTOG 85-01 determined the standard therapy of 50.4 Gy radiotherapy plus concurrent chemotherapy for locally advanced EC.^18^ Furthermore, the randomized RTOG INT 0123 trial compared chemoradiotherapy with high-dose (64.8 Gy/1.8 Gy) and standard-dose (50.4 Gy/1.8 Gy) combined with cisplatin/5-fluorouracil due to the high local failure rate, which once again consolidated the status of CCRT in the treatment of nonsurgical EC.^19,20^ The recent ARTDECO study reported that radiation dose of CCRT escalation up to 61.6 Gy for the primary tumor did not result in a significant increase in local control vs 50.4 Gy for locally advanced EC whether adenocarcinoma or squamous cell carcinoma, but the toxic effects of the high-dose group displayed an upward trend.^21^ Nevertheless, there is a lack of randomized studies comparing CCRT with chemotherapy alone for patients with SOESCC. The present study strictly controlled the baseline to minimize bias and indicated that patients who received CCRT have better ORR, DCR, PFS, and OS compared with patients who underwent chemotherapy alone. Subsequently, the same advantages were confirmed in the cohort after PSM. In addition, a 2-center retrospective study of oligometastatic EC showed that CCRT of all tumor lesions for patients with EC and less than 3 metastatic lesions could moderately prolong PFS but did not achieve a significant improvement in OS.^22^ One possible explanation is limited by a median follow-up of 23 months and the inclusion of 3% patients with adenocarcinoma, while the median follow-up time of this study was 37 months, and all patients had squamous cell carcinoma, which may be one of the reasons for superior PFS and OS in the CCRT group. In a single-institution study of patients with metachronous oligometastatic EC,^23^ the addition of local radiotherapy for all metastases was associated with better OS and can be used as an independent prognostic factor for patients. However, the PFS end point of the study was missing, and only 82 patients were enrolled. In contrast, our study included a large sample cohort and ensured the integrity of patient data. Moreover, another study on the prognostic model of oligometastatic EC^24^ also found that radiotherapy for a primary esophageal tumor was an independent protective factor for PFS and OS, and the local treatment of metastatic lesions was also associated with a preferable prognosis. Our study also found that CCRT for all tumor lesions was an independent prognostic factor for PFS and OS in patients with SOESCC.

A decision tree, an intuitive risk stratification model, has been successfully applied to some tumors.^25,26,27^ In our study, a recursive method was used to classify patients with SOESCC into different risk levels for progression and mortality. ROC and maximum AUC of decision trees for progression and mortality risk were 0.820 and 0.894, respectively, and the calibration curves displayed excellent calibration, which indicated that our models had highly accurate partition ability. Consequently, patients with high risk still had a high potential for progression and mortality after active initial treatment based on our decision trees, which suggests that it is necessary to intervene earlier with more intensive treatment to improve survival. Immune checkpoint inhibitors may be a promising route for these patients, and the consistent results of the ATTRACTION-3 study,^28^ ESCORT study^29^ and KEYNOTE-181 study^30^ showed that the anti–programmed cell death receptor 1 (PD-1) antibody was associated with a significant improvement in OS and a favorable safety profile in previously treated patients with advanced EC compared with chemotherapy and might represent a new standard second-line treatment option. Moreover, the recent KEYNOTE-590 study^31^ and ESCORT-1st study^32^ also found that the addition of anti–PD-1 antibody to chemotherapy significantly improved PFS and OS for patients with previously untreated advanced EC compared with chemotherapy alone, and has a manageable safety profile. Patients with intermediate and low risk based on the current treatment modality had a median PFS of greater than 11.4 (95% CI, 10.5-12.4) months and a median OS of greater than 23.6 (95% CI, 21.1-25.5) months in our decision trees. While the median PFS was 7.5 (95% CI, 6.2-8.2) months and 6.9 (95% CI, 5.8-7.4) months for KEYNOTE-590 and ESCORT-1st studies, respectively, and the median OS was 13.9 (95% CI, 11.1-17.7) months and 15.3 (95% CI, 12.8-17.3) months, respectively. Notably, the comparison of clinical outcomes between different treatment modalities still needs head-to-head study to control and minimize bias. Therefore, the application of anti–PD-1 antibody may be delayed in these patients, thereby reducing costs while achieving long-term survival. However, early individualized treatment with immune checkpoint inhibitors may have superior survival benefits for patients with high risk. Thus, our decision tree provides a new approach for the risk stratification of patients with SOESCC and could be used as a supplementary method for the tumor-node-metastasis staging system.

### Limitations

This study has limitations: the main limitation is the retrospective nature; thus, it is hypothesis-generating rather than confirmatory. As it is the first study we are aware of to investigate the potential application of a decision tree for patients with SOESCC in a modern context, the next logical steps should be to design a large sample prospective trial based on these partitions to further validate this prediction model.

## Conclusions

In this study, we found that CCRT as a first-line treatment for patients with SOESCC had superior survival and controllable toxic effects compared with chemotherapy alone. Patients with low risk had promising long-term survival based on the current treatment modality. The predictive information of our decision tree can be integrated into future clinical trials to provide accurate decision-making for the treatment of patients with SOESCC.

## Notes

Supplement 1. **eFigure 1.** Cox Multivariate Regression Analysis for Progression-Free Survival **eFigure 2.** Cox Multivariate Regression Analysis for Overall Survival **eFigure 3.** Standardized Absolute Mean Differences Before and After Propensity Score Matching **eFigure 4.** Performance Evaluation of the Decision Trees in the External Validation Cohort **eTable 1. **Baseline Characteristics Before and After Propensity Score Matching **eTable 2. **Characteristics of the Development and Validation Cohorts **eTable 3. **Factors for Generating Decision Trees **eTable 4. **Progression Risk Stratification of Decision Tree for Patients With SOESCC **eTable 5. **Mortality Risk Stratification of Decision Tree for Patients With SOESCC **eTable 6. **Grade 3 or Greater Toxic Effects by Group

Supplement 2.
Data Sharing Statement
