# Supplementary material for: Evaluation of Concurrent Chemoradiotherapy for Survival Outcomes in Patients With Synchronous Oligometastatic Esophageal Squamous Cell Carcinoma
Source: JAMA Netw Open. 2022 Dec 1;5(12):e2244619. doi: 10.1001/jamanetworkopen.2022.44619 (PMC9716398; doi:10.1001/jamanetworkopen.2022.44619)
Supplement: Supplement 1. — eFigure 1. Cox Multivariate Regression Analysis for Progression-Free Survival eFigure 2. Cox Multivariate Regression Analysis for Overall Survival eFigure 3. Standardized Absolute Mean Differences Before and After Propensity Score Matching eFigure 4. Performance Evaluation of the Decision Trees in the External Validation Cohort eTable 1. Baseline Characteristics Before and After Propensity Score Matching eTable 2. Characteristics of the Development and Validation Cohorts eTable 3. Factors for Generating Decision Trees eTable 4. Progression Risk Stratification of Decision Tree for Patients With SOESCC eTable 5. Mortality Risk Stratification of Decision Tree for Patients With SOESCC eTable 6. Grade 3 or Greater Toxic Effects by Group [file jamanetwopen-e2244619-s001.pdf]

Supplemental Online Content

Shi Z, Zhu X, Ruan C, et al. Evaluation of concurrent chemoradiotherapy for survival outcomes in patients with synchronous oligometastatic esophageal squamous cell carcinoma. *JAMA Netw Open.* 2022;5(12):e2244619.
doi:10.1001/jamanetworkopen.2022.44619

eFigure 1. Cox Multivariate Regression Analysis for Progression-Free Survival

eFigure 2. Cox Multivariate Regression Analysis for Overall Survival

eFigure 3. Standardized Absolute Mean Differences Before and After Propensity Score Matching

eFigure 4. Performance Evaluation of the Decision Trees in the External Validation Cohort

eTable 1. Baseline Characteristics Before and After Propensity Score Matching

eTable 2. Characteristics of the Development and Validation Cohorts

eTable 3. Factors for Generating Decision Trees

eTable 4. Progression Risk Stratification of Decision Tree for Patients With SOESCC

eTable 5. Mortality Risk Stratification of Decision Tree for Patients With SOESCC

eTable 6. Grade 3 or Greater Toxic Effects by Group

This supplemental material has been provided by the authors to give readers additional information about their work.

© 2022 Shi Z et al. *JAMA Network Open.*

eFigure 1. Cox Multivariate Regression Analysis for Progression-Free Survival

Characteristic	Subgroup	HR (95% CI)	*P* value
Age	<60 years	reference	
	≥60 years	0.715 (0.586-0.871)	0.001
Sex	male	reference	
	female	0.949 (0.775-1.161)	0.611
ECOG PS	0	reference	
	1	1.848 (1.312-2.602)	0.000
	2	1.779 (1.180-2.683)	0.006
Primary tumor length	<5 cm	reference	
	≥5 cm	0.878 (0.726-1.063)	0.183
Primary tumor location	cervical	reference	
	upper thoracic	1.273 (0.800-2.025)	0.308
	middle thoracic	1.324 (0.874-2.005)	0.186
	lower thoracic	1.468 (0.950-2.268)	0.084
No. of metastatic organs	1	reference	
	2-3	0.943 (0.657-1.354)	0.750
No. of metastatic lesions	1-3	reference	
	4-5	1.249 (0.873-1.789)	0.224
Chemotherapy regimen	DP	reference	
	PF	1.136 (0.944-1.368)	0.176
Treatment modality	CT alone	reference	
	CRT	0.685 (0.565-0.832)	0.000

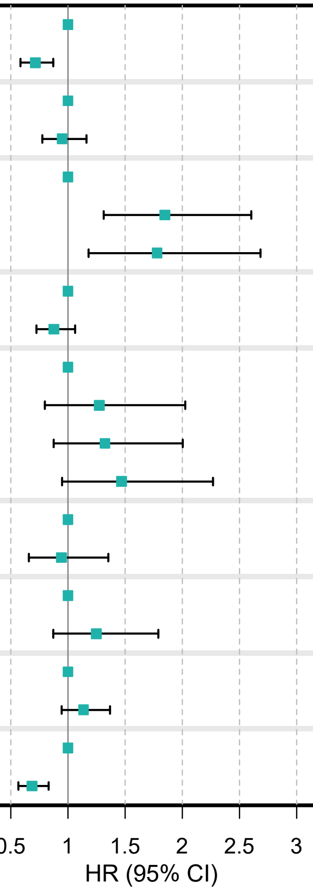

Abbreviations: ECOG, Eastern Cooperative Oncology Group; PS, Performance Status; DP, cisplatin plus docetaxel; PF, cisplatin plus 5-fluorouracil; CT alone, chemotherapy alone; CRT, concurrent chemoradiotherapy; HR, hazard ratio; CI, confidence interval.

© 2022 Shi Z et al. *JAMA Network Open.*

eFigure 2. Cox Multivariate Regression Analysis for Overall Survival

Characteristic	Subgroup	HR (95% CI)	*P* value
Age	<60 years	reference	
	≥60 years	0.715 (0.575-0.889)	0.003
Sex	male	reference	
	female	0.875 (0.699-1.094)	0.241
ECOG PS	0	reference	
	1	2.543 (1.689-3.828)	0.000
	2	2.868 (1.784-4.613)	0.000
Primary tumor length	<5 cm	reference	
	≥5 cm	0.932 (0.755-1.149)	0.509
Primary tumor location	cervical	reference	
	upper thoracic	1.504 (0.916-2.470)	0.107
	middle thoracic	1.265 (0.809-1.979)	0.302
	lower thoracic	1.396 (0.872-2.236)	0.165
No. of metastatic organs	1	reference	
	2-3	0.885 (0.603-1.300)	0.534
No. of metastatic lesions	1-3	reference	
	4-5	1.492 (1.018-2.185)	0.040
Chemotherapy regimen	DP	reference	
	PF	1.093 (0.893-1.338)	0.389
Treatment modality	CT alone	reference	
	CRT	0.750 (0.607-0.926)	0.008

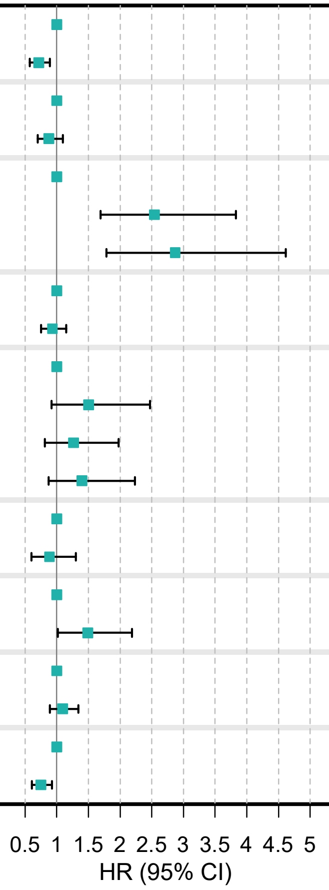

Abbreviations: ECOG, Eastern Cooperative Oncology Group; PS, Performance Status; DP, cisplatin plus docetaxel; PF, cisplatin plus 5-fluorouracil; CT alone, chemotherapy alone; CRT, concurrent chemoradiotherapy; HR, hazard ratio; CI, confidence interval.

© 2022 Shi Z et al. *JAMA Network Open.*

eFigure 3. Standardized Absolute Mean Differences Before and After Propensity Score Matching

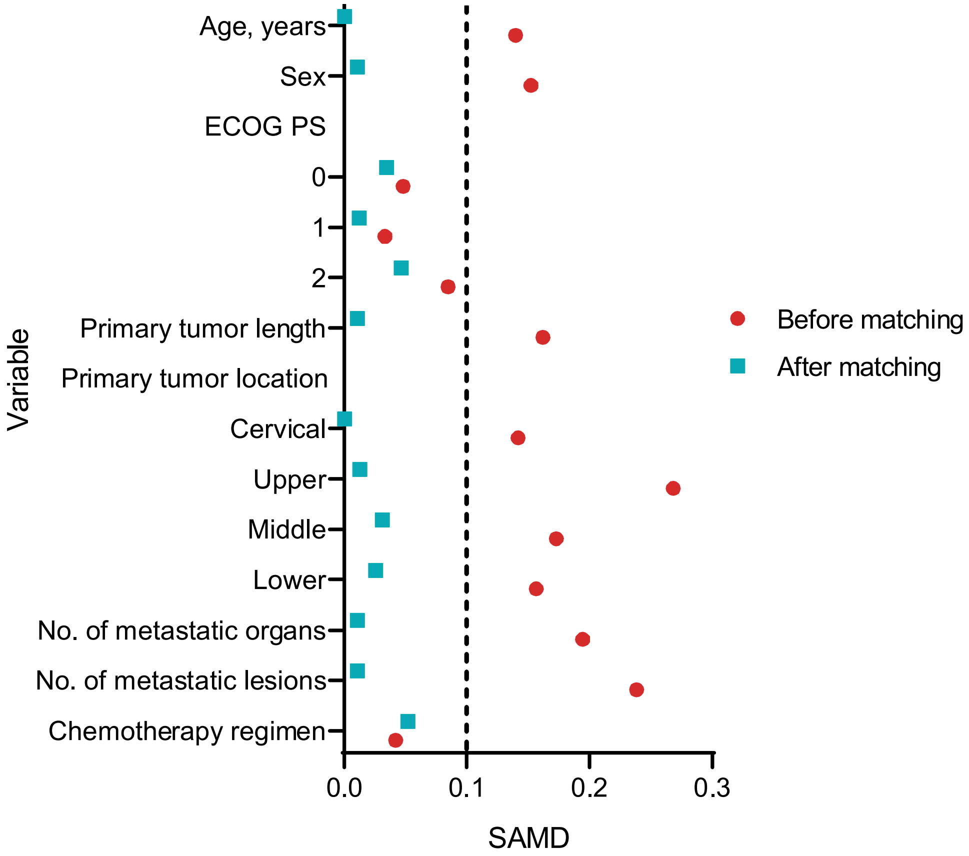

Abbreviations: ECOG, Eastern Cooperative Oncology Group; PS, Performance Status; SAMD, standardized absolute mean differences.

© 2022 Shi Z et al. *JAMA Network Open.*

eFigure 4. Performance Evaluation of the Decision Trees in the External Validation Cohort

Cumulative progression (A) and mortality (B) risk stratification of decision trees. The calibration curves to predict progression (C) and mortality (D).

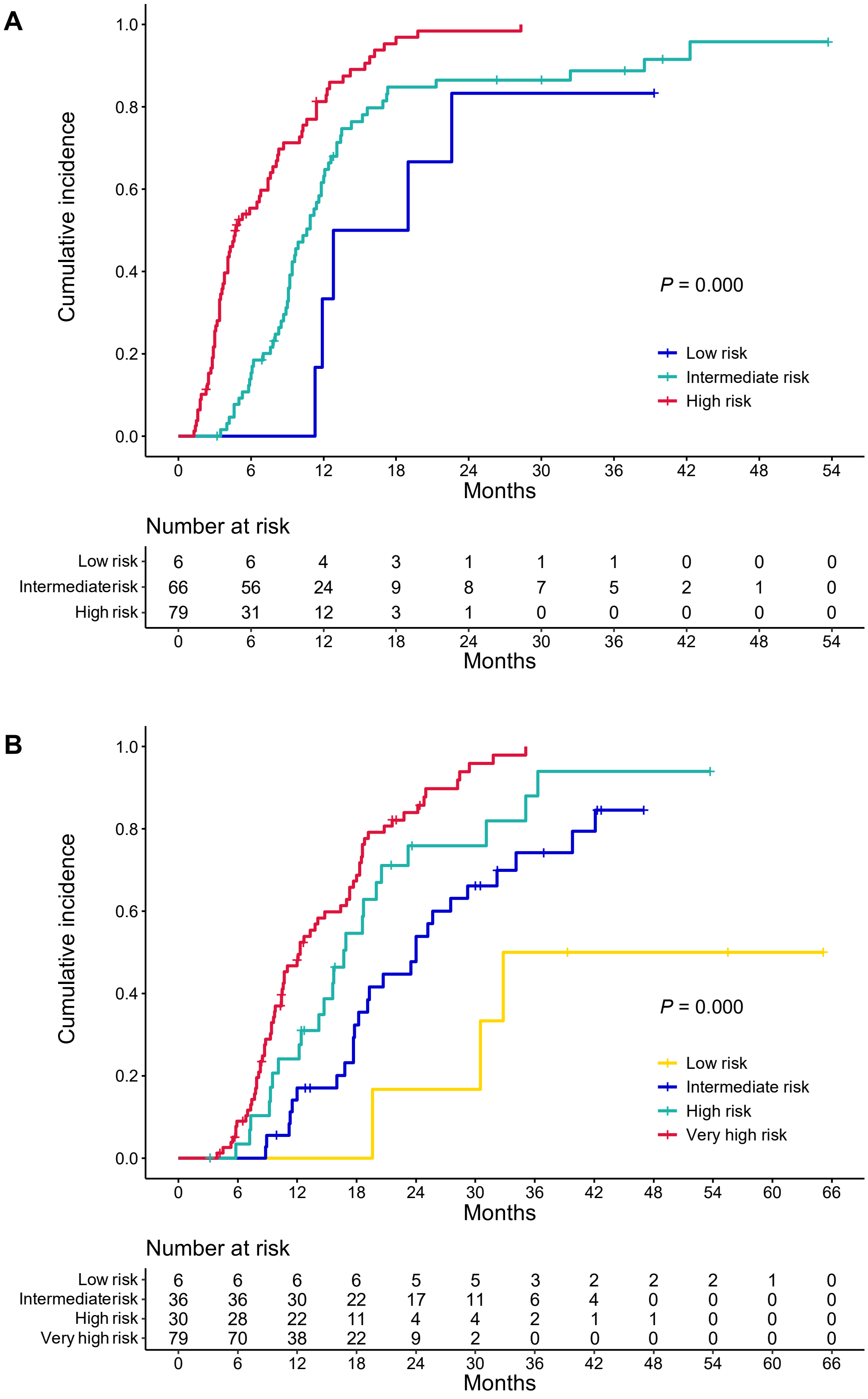

© 2022 Shi Z et al. *JAMA Network Open.*

C

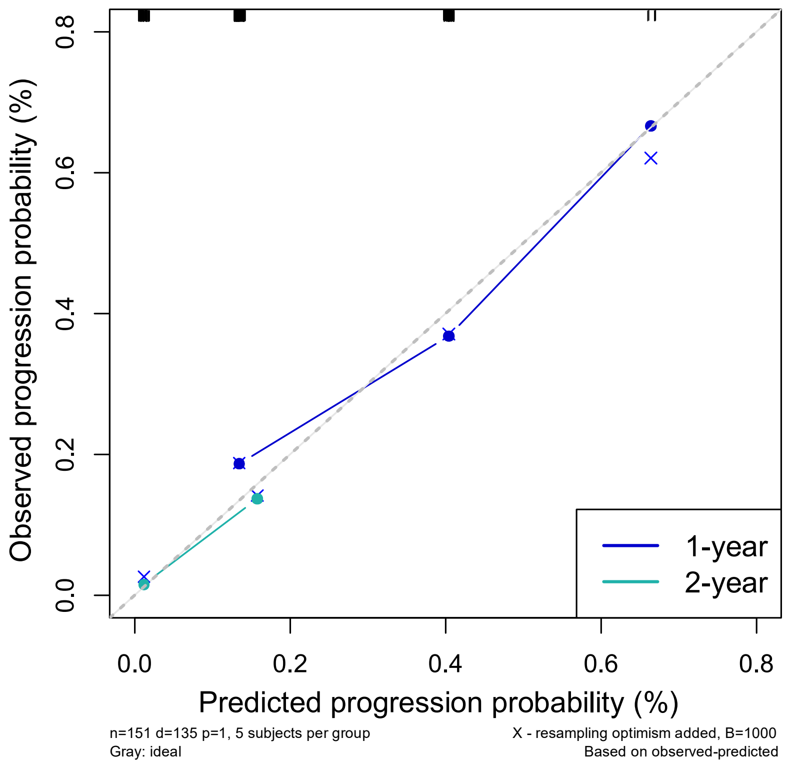

D

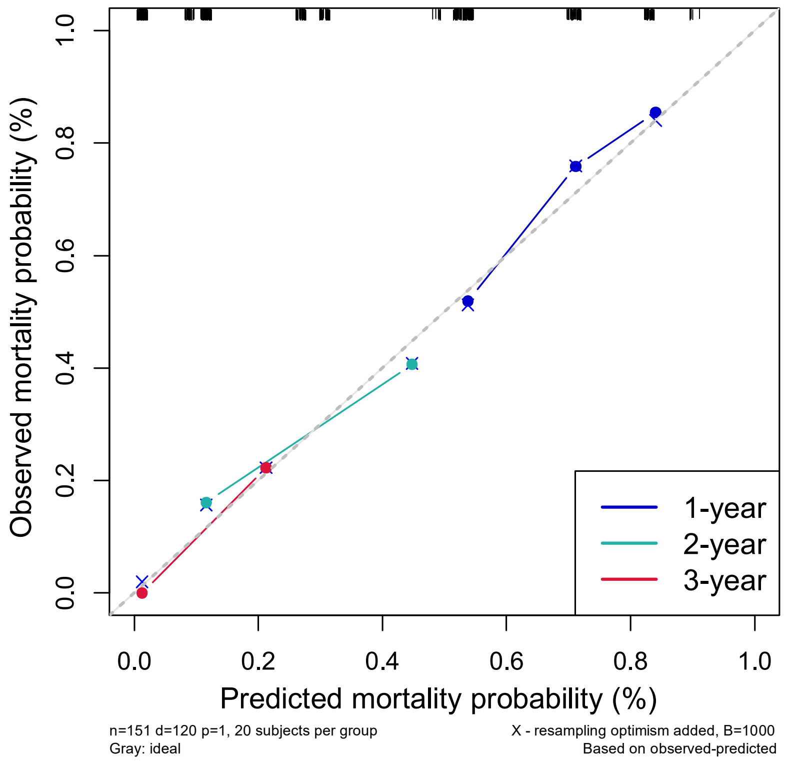

© 2022 Shi Z et al. *JAMA Network Open.*

eTable 1. Baseline Characteristics Before and After Propensity Score Matching

Characteristic	Before matching				After matching		
	CT alone (n=292)	CRT (n=240)	SAMD		CT alone (n=193)	CRT (n=193)	SAMD
**Age, years (%)**							
<60	105 (36.0)	71 (29.6)	0.140		58 (30.1)	58 (30.1)	0.000
≥60	187 (64.0)	169 (70.4)	0.140		135 (69.9)	135 (69.9)	0.000
**Sex (%)**							
Male	211 (72.3)	156 (65.0)	0.152		130 (67.4)	131 (67.9)	0.011
Female	81 (27.7)	84 (35.0)	0.152		63 (32.6)	62 (32.1)	0.011
**ECOG PS (%)**							
0	25 (8.6)	24 (10.0)	0.048		16 (8.3)	18 (9.3)	0.035
1	221 (75.7)	185 (77.1)	0.033		149 (77.2)	150 (77.7)	0.012
2	46 (15.8)	31 (12.9)	0.085		28 (14.5)	25 (13.0)	0.046
**Primary tumor length (%)**							
<5 cm	126 (43.2)	85 (35.4)	0.162		74 (38.3)	75 (38.9)	0.011
≥5 cm	166 (56.8)	155 (64.6)	0.162		119 (61.7)	118 (61.1)	0.011
**Primary tumor location (%)**							
Cervical	11 (3.8)	18 (7.5)	0.142		10 (5.2)	10 (5.2)	0.000
Upper	33 (11.3)	54 (22.5)	0.268		31 (16.1)	32 (16.6)	0.012
Middle	170 (58.2)	119 (49.6)	0.173		110 (57.0)	107 (55.4)	0.031
Lower	78 (26.7)	49 (20.4)	0.156		42 (21.8)	44 (22.8)	0.026
**No. of metastatic organs (%)**							
1	164 (56.2)	157 (65.4)	0.195		124 (64.2)	123 (63.7)	0.011
2-3	128 (43.8)	83 (34.6)	0.195		69 (35.8)	70 (36.3)	0.011

© 2022 Shi Z et al. *JAMA Network Open.*

**No. of metastatic lesions (%)**							
1-3	154 (52.7)	154 (64.2)	0.238		120 (62.2)	119 (61.7)	0.011
4-5	138 (47.3)	86 (35.8)	0.238		73 (37.8)	74 (38.3)	0.011
**Chemotherapy regimen (%)**							
DP	146 (50.0)	115 (47.9)	0.042		95 (49.2)	90 (46.6)	0.052
PF	146 (50.0)	125 (52.1)	0.042		98 (50.8)	103 (53.4)	0.052

Abbreviations: CT alone, chemotherapy alone; CRT, concurrent chemoradiotherapy; SAMD, standardized absolute mean differences; ECOG, Eastern Cooperative Oncology Group; PS, Performance Status; DP, cisplatin plus docetaxel; PF, cisplatin plus 5-fluorouracil.

© 2022 Shi Z et al. *JAMA Network Open.*

eTable 2. Characteristics of the Development and Validation Cohorts

Characteristic	Development Cohort				Validation Cohort		
	CT alone (n=202)	CRT (n=179)	*P* Value		CT alone (n=90)	CRT (n=61)	*P* Value
**Age, years (%)**							
<60	73 (36.1)	54 (30.2)	0.217		32 (35.6)	17 (27.9)	0.322
≥60	129 (63.9)	125 (69.8)			58 (64.4)	44 (72.1)	
**Sex (%)**							
Male	150 (74.3)	119 (66.5)	0.096		61 (67.8)	37 (60.7)	0.368
Female	52 (25.7)	60 (33.5)			29 (32.2)	24 (39.3)	
**ECOG PS (%)**							
0	17 (8.4)	20 (11.2)	0.216		8 (8.9)	4 (6.6)	0.896
1	152 (75.2)	136 (76.0)			69 (76.7)	49 (80.3)	
2	33 (16.3)	23 (12.8)			13 (14.4)	8 (13.1)	
**Primary tumor length (%)**							
<5 cm	86 (42.6)	65 (36.3)	0.212		40 (44.4)	20 (32.8)	0.151
≥5 cm	116 (57.4)	114 (63.7)			50 (55.6)	41 (67.2)	
**Primary tumor location (%)**							
Cervical	7 (3.5)	15 (8.4)	0.004		4 (4.4)	3 (4.9)	0.093
Upper	23 (11.4)	39 (21.8)			10 (11.1)	15 (24.6)	
Middle	122 (60.4)	87 (48.6)			48 (53.3)	32 (52.5)	
Lower	50 (24.8)	38 (21.2)			28 (31.1)	11 (18.0)	
**No. of metastatic organs (%)**							
1	118 (58.4)	113 (63.1)	0.347		46 (51.1)	44 (72.1)	0.010
2-3	84 (41.6)	66 (36.9)			44 (48.9)	17 (27.9)	
**No. of metastatic**							

© 2022 Shi Z et al. *JAMA Network Open.*

**lesions (%)**							
1-3	113 (55.9)	109 (60.9)	0.328		41 (45.6)	45 (73.8)	0.001
4-5	89 (44.1)	70 (39.1)			49 (54.4)	16 (26.2)	
**Chemotherapy regimen (%)**							
DP	101 (50.0)	82 (45.8)	0.414		45 (50.0)	33 (54.1)	0.621
PF	101 (50.0)	97 (54.2)			45 (50.0)	28 (45.9)	
**Tumor response (%)**							
CR	5 (2.5)	22 (12.3)	0.000		1 (1.1)	7 (11.5)	0.011
PR	78 (38.6)	85 (47.5)			39 (43.3)	25 (41.0)	
SD	47 (23.3)	44 (24.6)			22 (24.4)	19 (31.1)	
PD	72 (35.6)	28 (15.6)			28 (31.1)	10 (16.4)	

Abbreviations: CT alone, chemotherapy alone; CRT, concurrent chemoradiotherapy; ECOG, Eastern Cooperative Oncology Group; PS, Performance Status; DP, cisplatin plus docetaxel; PF, cisplatin plus 5-fluorouracil; CR, complete response; PR, partial response; SD, stable disease; PD, progressive disease.

© 2022 Shi Z et al. *JAMA Network Open.*

eTable 3. Factors for Generating Decision Trees

Variable	Progression-free survival				Overall survival		
	HR	95% CI	*P* Value		HR	95% CI	*P* Value
Age (<60 vs. ≥60 years)	0.640	0.509-0.805	0.000		0.673	0.522-0.869	0.002
Sex (male vs. female)	0.957	0.759-1.208	0.714		0.847	0.651-1.103	0.217
ECOG PS (0 vs. 1-2)	2.291	1.558-3.366	0.000		2.639	1.658-4.199	0.000
Primary tumor length (<5 vs. ≥5cm)	0.809	0.651-1.005	0.056		0.880	0.690-1.122	0.301
Primary tumor location (cervical/upper thoracic vs. middle/lower thoracic)	1.398	1.075-1.819	0.012		1.202	0.901-1.603	0.211
No. of metastatic organs (1 vs. 2-3)	1.334	1.071-1.661	0.010		1.302	1.019-1.662	0.035
No. of metastatic lesions (1-3 vs. 4-5)	1.400	1.128-1.737	0.002		1.427	1.122-1.815	0.004
Chemotherapy regimen (DP vs. PF)	1.051	0.850-1.300	0.645		1.025	0.809-1.299	0.836
Treatment modality (CT alone vs. CRT)	0.636	0.513-0.788	0.000		0.697	0.548-0.886	0.003
Tumor Response (CR/PR vs. SD/PD)	3.219	2.575-4.024	0.000		3.490	2.706-4.501	0.000

Abbreviations: HR, hazard ratio; CI, confidence interval; ECOG, Eastern Cooperative Oncology Group; PS, Performance Status; DP, cisplatin plus docetaxel; PF, cisplatin plus 5-fluorouracil; CT alone, chemotherapy alone; CRT, concurrent chemoradiotherapy; CR, complete response; PR, partial response; SD, stable disease; PD, progressive disease.

© 2022 Shi Z et al. *JAMA Network Open.*

eTable 4. Progression Risk Stratification of Decision Tree for Patients With SOESCC

Risk stratification	Clinical parameters		No. patients (No. events)	Median PFS (months)	Risk of progression
	Tumor response	ECOG PS			
Low risk	CR/PR	0	18 (24)	24.3	0.35
Intermediate risk	CR/PR	1-2	149 (166)	11.4	0.75
High risk	SD/PD	/	176 (191)	4.9	1.9

Abbreviations: CR, complete response; PR, partial response; SD, stable disease; PD, progressive disease; ECOG, Eastern Cooperative Oncology Group; PS, Performance Status; PFS, progression-free survival.

© 2022 Shi Z et al. *JAMA Network Open.*

eTable 5. Mortality Risk Stratification of Decision Tree for Patients With SOESCC

Risk stratification	Clinical parameters			No. patients (No. events)	Median OS (months)	Risk of mortality
	Tumor response	ECOG PS	No. metastatic lesions			
Low risk	CR/PR	0	/	10 (24)	59.7	0.26
Intermediate risk	CR/PR	1-2	1-3	71 (104)	23.6	0.66
High risk	CR/PR	1-2	4-5	54 (62)	19.3	0.99
Very high risk	SD/PD	/	/	143 (191)	11.7	2

Abbreviations: CR, complete response; PR, partial response; SD, stable disease; PD, progressive disease; ECOG, Eastern Cooperative Oncology Group; PS, Performance Status; OS, overall survival.

© 2022 Shi Z et al. *JAMA Network Open.*

eTable 6. Grade 3 or Greater Toxic Effects by Group

Toxicities	CT alone (n=292)			CRT (n=240)		*P* Value
	Grade 3	Grade 4		Grade 3	Grade 4	
Leukocytopenia (%)	47 (16.1)	18 (6.2)		51 (21.3)	23 (9.6)	0.025
Nausea/Vomiting (%)	29 (9.9)	0 (0)		27 (11.3)	0 (0)	0.622
Fatigue (%)	10 (3.4)	0 (0)		11 (4.6)	0 (0)	0.495
ALT elevation (%)	12 (4.1)	0 (0)		9 (3.8)	0 (0)	0.832
Radiation pneumonitis (%)	-	-		13 (5.4)	3 (1.3)	-
Radiation esophagitis (%)	-	-		15 (6.3)	2 (0.8)	-

Abbreviations: CT alone, chemotherapy alone; CRT, concurrent chemoradiotherapy; ALT, alanine aminotransferase.

© 2022 Shi Z et al. *JAMA Network Open.*
