# Supplementary material for: Evaluation of Concurrent Chemoradiotherapy for Survival Outcomes in Patients With Synchronous Oligometastatic Esophageal Squamous Cell Carcinoma
Source: JAMA Netw Open. 2022 Dec 1;5(12):e2244619. doi: 10.1001/jamanetworkopen.2022.44619 (PMC9716398; doi:10.1001/jamanetworkopen.2022.44619)
Supplement: Supplement 2. — Data Sharing Statement [file jamanetwopen-e2244619-s002.pdf]

Data Sharing Statement

Shi. Evaluation of Concurrent Chemoradiotherapy for Survival Outcomes in Patients With Synchronous Oligometastatic Esophageal Squamous Cell Carcinoma. *JAMA Netw Open*. Published December 01, 2022. doi:10.1001/jamanetworkopen.2022.44619

Data
Data available: Yes
Data types: Deidentified participant data
How to access data: yongshun2007@163.com
When available: With publication

Supporting Documents
Document types: Statistical/analytic code
How to access documents: yongshun2007@163.com
When available: With publication

Additional Information
Who can access the data: researchers whose proposed use of the data has been approved
Types of analyses: specified purpose
Mechanisms of data availability: signed data access agreement
